# A generalized quantitative antibody homeostasis model: maintenance of global antibody equilibrium by effector functions

**DOI:** 10.1038/cti.2017.50

**Published:** 2017-11-17

**Authors:** József Prechl

**Affiliations:** 1 R&D Laboratory, Diagnostcum zrt, Budapest, Hungary

## Abstract

The homeostasis of antibodies can be characterized as a balanced production, target-binding and receptor-mediated elimination regulated by an interaction network, which controls B-cell development and selection. Recently, we proposed a quantitative model to describe how the concentration and affinity of interacting partners generates a network. Here we argue that this physical, quantitative approach can be extended for the interpretation of effector functions of antibodies. We define global antibody equilibrium as the zone of molar equivalence of free antibody, free antigen and immune complex concentrations and of dissociation constant of apparent affinity: [Ab]=[Ag]=[AbAg]=*K*_D_. This zone corresponds to the biologically relevant *K*_D_ range of reversible interactions. We show that thermodynamic and kinetic properties of antibody–antigen interactions correlate with immunological functions. The formation of stable, long-lived immune complexes correspond to a decrease of entropy and is a prerequisite for the generation of higher-order complexes. As the energy of formation of complexes increases, we observe a gradual shift from silent clearance to inflammatory reactions. These rules can also be applied to complement activation-related immune effector processes, linking the physicochemical principles of innate and adaptive humoral responses. Affinity of the receptors mediating effector functions shows a wide range of affinities, allowing the continuous sampling of antibody-bound antigen over the complete range of concentrations. The generation of multivalent, multicomponent complexes triggers effector functions by crosslinking these receptors on effector cells with increasing enzymatic degradation potential. Thus, antibody homeostasis is a thermodynamic system with complex network properties, nested into the host organism by proper immunoregulatory and effector pathways. Maintenance of global antibody equilibrium is achieved by innate qualitative signals modulating a quantitative adaptive immune system, which regulates molecular integrity of the host by tuning the degradation and recycling of molecules from silent removal to inflammatory elimination.

## Introduction

The functioning of the humoral adaptive immune system relies on the generation and maintenance of an elaborate and complex system of binding molecules called antibodies. The vast number of target molecules and breadth of binding affinities combined with the dynamic, evolving nature of antibody (Ab) production renders the system capable of adapting to complex changing molecular environments but makes quantitative systems biological description difficult. We proposed a generalized quantitative model for the homeostatic function of clonal humoral immune system, mapping antibody–antigen (Ag) interaction network in a virtual physical space. In our model we assumed that the essence of antibody homeostasis is the maintenance of a regulated balance between the saturation of B-cell receptors (BCR) by antigen^1^ and the saturation of antigen by antibodies, thereby controlling B-cell development and antigen fate in the body.^2^ Regulated antibody production drives the system of molecules towards a binding equilibrium, which characterizes the nature and outcome of immunological recognition. The formation of antibody–antigen immune complexes (AbAg) is followed by their removal from the body. In this third paper, we describe how physical properties of two distinct but cooperative binding events, namely the binding antibody to antigen targets and antibody-mediated immune complex binding to Fc receptors, further expands and modulates the network of antibody interactions, empowering the immune system with the control of the rate and mode of molecular degradation and reuse. We shall argue that our quantitative model supplements qualitative molecular approaches to immune function and provides a general physical framework with simple straightforward rules of operation.

## Global antibody equilibrium

**Figure 1:**
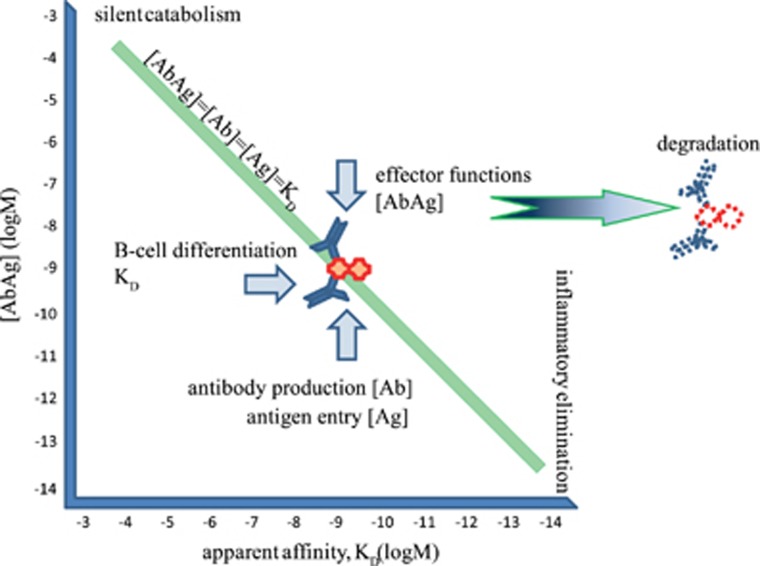
Equilibrium at equimolar antibody and antigen concentrations—the global antibody equilibrium. For each *K*_D_ value there is one special condition, when concentration of free and bound forms of antibody and antigen are all equal. For the complete range of immunologically relevant *K*_D_s and all interacting partners in the system, the existence of a global equilibrium we call Global Antibody Equilibrium. The function of the humoral adaptive system is the maintenance of this equilibrium by adjusting *K*_D_ and [Ab] by B-cell differentiation and proliferation and [AbAg] by effector functions leading to degradation. This leads to the regulation of [Ag] according to its quality and quantity. Blue arrows indicate the direction of change of the relevant parameter.

We have defined humoral adaptive immunity as a system where qualitative signals of antigenic molecules adjust equilibrium dissociation constant *K*_D_ to be equal to free antigen concentration [Ag] via regulation of B-cell selection and differentiation.^1^ Quantitative signals drive B-cell proliferation and antibody production to adjust free antibody concentration [Ab]=*K*_D_ to achieve relevant antigen saturation.^2^ Therefore, the system tends to reach *K*_D_=[Ag]=[Ab] at which point [AbAg] will also be equal to *K*_D_ (Figure 1) based on the general equation:


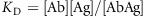


Thus, we can think of the clonal humoral immune system as one that controls [AbAg] by adjusting *K*_D_ and [Ab] according to changing [Ag] and the quality of antigen:


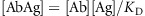


In turn this provides a means of controlling the rate and efficiency of removal of antigen from the host. Since the constant production of antibody and the continuous presence and appearance of antigen perpetually generates immune complexes, the only way of keeping [AbAg] constant is to remove it from the system. Immune complex removal is achieved by effector functions coupled to the humoral immune system—the rate, efficiency and quality of removal are key characteristics of, and basically define immune responsiveness and responses, as we shall see in the following sections.

## Physical aspects of immune complex formation

### Affinity and avidity

**Figure 2:**
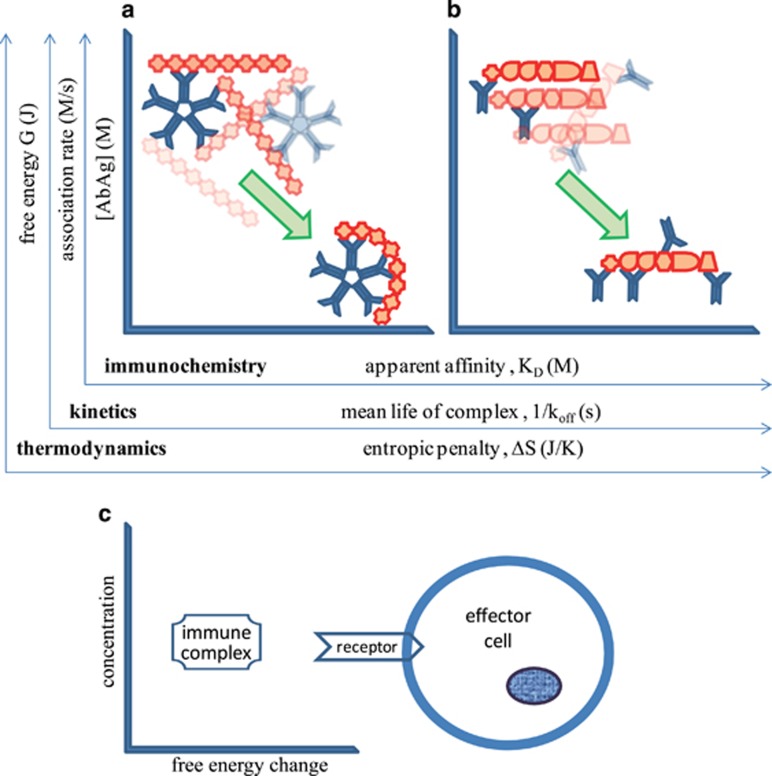
Interpretation and correlation of biophysical aspects of antibody–antigen interactions. Interactions of a single pentameric IgM (**a**) and several dimeric IgG (**b**) molecules are used as models. The same events can be interpreted from immunochemical, kinetic and thermodynamic aspects. Immune responses are characterized by 1) increased avidity—multivalent interactions between antibody and antigen result in higher apparent affinity; (2) altered kinetics—the length of the interaction also increases with increased avidity; and (3) different thermodynamic properties—high avidity interactions are characterized by higher free energy loss and greater decrease in entropy of the interacting molecules. (**c**) Interaction of the complexes with cells is mediated by receptors of varying affinities, in turn cellular effects are determined by the extent of receptor crosslinkage and the identity of the effector cell.

Antibody molecules bind to their targets via the paratope: a surface formed by the hypervariable peptide loops responsible for binding. The paratope contacts the epitope, the binding surface of the antigen, and the binding can be characterized by the affinity of the interaction. Multiple identical binding sites of antibodies can simultaneously bind to a multivalent target, carrying multiple identical epitopes. Recognition of a target with repetitive patterns is therefore promoted by placing several binding sites onto an antibody. Each site contributes to the cumulative binding strength, called avidity. IgM, the first antibody produced by a B cell, is pentameric or hexameric, with 10 or 12 paratopes, respectively, allowing a significant increase in avidity upon multiple engagement (Figure 2). For the sake of consistency, we will continue to use the expression 'apparent affinity' as a general term for the characterization of the strength of molecular interactions, and introduce change in free energy as a dimension of measurement (see below).

In addition to the immunochemical interpretation *K*_D_= [Ab][Ag]/[AbAg], equilibrium can also be approached from the thermodynamic or from the kinetic aspect. Appreciation of these aspects helps a better understanding of immunological effector functions (Figure 2).

### Kinetics

The association rate constant *k*_on_ of the interacting molecules along with the dissociation rate constant *k*_off_ together describe where an equilibrium between free and bound forms of a particular pair of antibody and antigen is established, defined by the equation


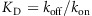


As suggested by the equation, the same *K*_D_ can be the result of very fast and very slow event rates as long as their ratio remains constant. The association rate constant has an upper limit due to molecular movements being restricted by diffusion.^3^ If we assume that *k*_on_ is similar in the complete *K*_D_ range then the rate of association (rate=*k*_on_[Ab][Ag]) will be determined by the concentration of the interacting molecules. High concentrations of interacting antibody and antigen thus lead to frequent binding events (Figures 2a and b; kinetics). We can also assume that low-affinity interactions are characterized by fast off rates, which at high concentrations of interactors translate to high frequency on-off reactions. High-affinity interactions, on the other hand, are characterized by low dissociation rate constants, and low immune complex concentrations also result in low dissociation rates (rate=*k*_off_[AbAg]), leading to the generation of long-lasting complexes. Long-lived complexes have a higher chance of engaging in additional interactions both with other antibodies or receptors on effector cells.

### Thermodynamics

Spontaneous reactions are accompanied by a decrease of free energy of the reactants.^4^ The relationship between affinity and the change in free energy is given by the equation:


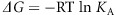


where Δ*G* stands for free energy change, *R* is molar gas constant (*R*=8.314 J mol^−1 ^K^−1^), *T* is thermodynamic temperature in Kelvins and *K*_A_ is the association rate constant. At constant temperature free energy change is thus proportional to affinity of the interaction: the greater the apparent affinity the greater the change in free energy. The advantage of using Δ*G* is that the formation of multicomponent complexes can also be characterized. This change of free energy has enthalpic (Δ*H*) and entropic (Δ*S*) components


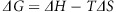


which can be used to describe the energy landscape of the antibody interaction network.^5^ Interacting molecules 'pay' for becoming part of a complex by losing entropy, here interpreted as freedom to change conformation; the 'fee' is called entropic penalty.

In the next sections, we will keep describing immune complex formation using concentration and free energy change (Figure 2c), because we will be leaving the range of reversible bimolecular interactions. One reason for that is the formation of higher-order complexes with multiple sites of a single antibody binding (Figure 2a) or multiple antibodies binding (Figure 2b), the other reason being the appearance of covalent binding as we shall see in the next sections.

## FcR-mediated effector functions

**Figure 3:**
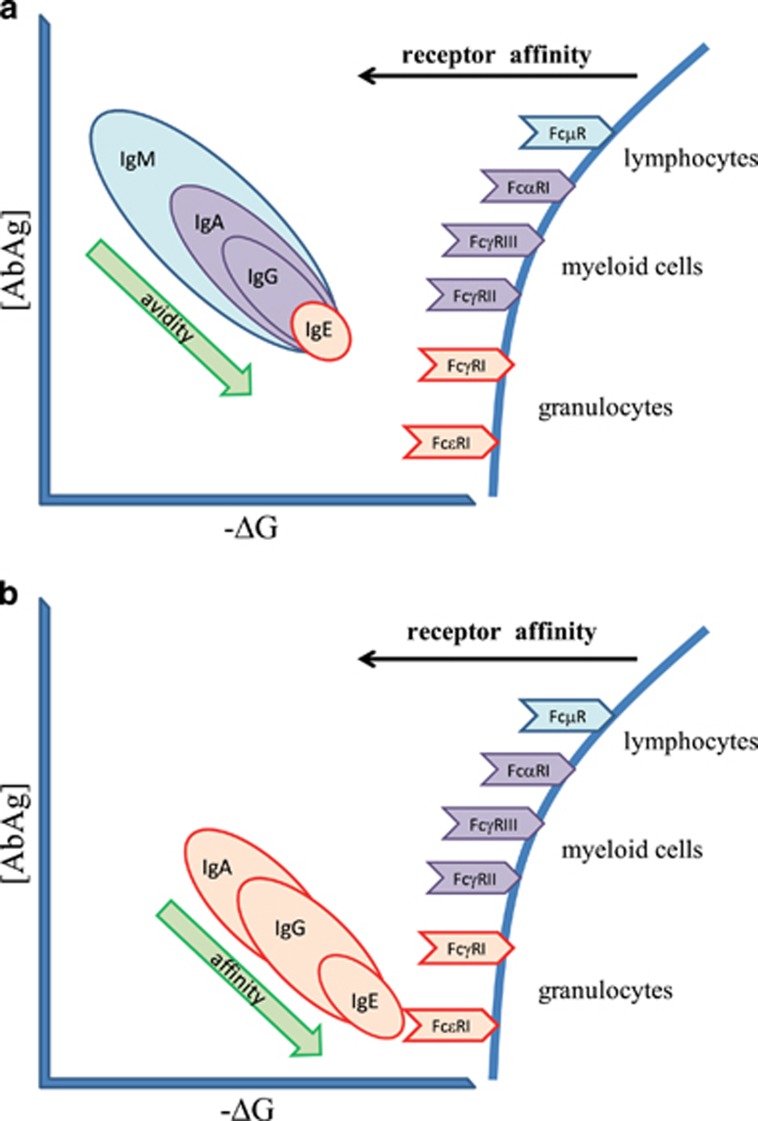
Effector functions of antibodies produced during TI and TD responses. (**a**) Thymus-independent antibody responses operate by exploiting avidity effects: clones selected by specificity can increase their apparent affinity by multivalent binding to the target, if the target carries multiple identical epitopes. Switching isotypes enhance cellular responses by binding to receptors with increasing affinity. (**b**) Thymus-dependent responses exploit affinity maturation besides isotype switching. Increased affinity to target is accompanied by the usage of isotypes with stronger effector functions. Affinity of IgE to FcεRI is so high that cellular receptors are decorated with monomeric IgE. The presence of multiple high-affinity antibodies on the target results in potent receptor crosslinkage, triggering robust cellular responses even via otherwise low-affinity receptors.

Effector functions of antibodies are exerted via interactions with cells. Neutralization might be regarded as an exemption, the neutralizing antibody binding to biologically important epitopes and interfering with their binding. However, even in this case complexes of the antigen and antibody need to be removed from the body at the end. Complement-dependent cytotoxicity is another exemption, which we discuss in the next section. Other effector functions, such as endocytosis, opsonin-mediated phagocytosis, frustrated phagocytosis, antibody-dependent cytotoxicity, complement-dependent phagocytosis, all depend on interactions of immune complexes with effector cells. Primary mediators of antibody–cell interactions are the receptors binding the Fc region of immunoglobulins, FcR.^6^ These are characterized by their specificity for each antibody class and by their affinities to various antibody isotypes. Here we consider FcR comprising one or more extracellular domains responsible for Fc binding that belong to the immunoglobulin superfamily (Figure 3). Reported affinities for FcμR^7^ and FcμαR^8^ are higher than suggested here, a discrepancy possibly caused by avidity effects and the use of transfected cells for affinity estimations. Nevertheless, our theoretical estimations need experimental confirmation. Affinities of FcαRI,^9^ FcγRs^10^ and FcεRI^11, 12^ have been extensively studied, and while IgG subclasses and glycoforms introduce further level of complexity,^10, 13, 14^ a general gradual increase of affinity in this order is observed as shown in Figure 3.

When considering the interactions of immune complexes with cells again, we have to take into account not only the affinity but also the valency and avidity of the interactions. Binding to multiple receptors by the same complex will crosslink receptors leading to enhanced signaling and more robust cellular responses. Monovalent engagement leads to homeostatic recycling in contrast to multivalent engagement, which can trigger cell activation via the FcR γ-chain.^9, 15, 16, 17^

Since we previously separately addressed thymus-independent (TI) and thymus-dependent (TD) antibody production,^2^ here we will also discuss effector functions using this categorization. We need to keep in mind, however, that these events actually show an overlap and continuity when represented in our molecular interaction space (Figures 3a and b).

### TI responses

In the absence of T-cell-derived co—stimulation B cells cannot initiate germinal center formation and cannot increase antibody affinity to target. TI responses therefore operate by taking advantage of avidity increase via multiple interactions, by changing antibody isotype and by adjusting antibody concentration (Figure 3a). IgM, the isotype displayed first by developing B cells is secreted in pentameric or hexameric form,^18^ the monomeric units covalently bound by interchain disulfide bridges or by the J-chain.^19^ Polymeric IgM molecules have therefore 10 to 12 antigen binding sites. Coupled with the flexibility of the molecule this results in multiple interactions with targets carrying repetitive epitopes. Such molecules are carbohydrates, glycoproteins, DNA and RNA, in addition to various microbial substances.^20, 21^ Free and antigen-bound IgM molecules attached to FcμR are endocytosed and shuttled to the lysosomes for degradation.^22^ Dimers of IgA form by J-chain incorporation, leading to tetravalent binding potential. Dimeric and monomeric IgA binds to CD89, also called FcαRI, which mediates phagocytosis and cellular activation.^23^ Priming of myeloid cells via IgG-mediated activation modulates FcαRI effects, a synergism potentially leading to inflammatory reactions.^24^ IgG2 is the characteristic isotype of responses against capsular bacteria.^13^ It has low affinity to most FcγRs, yet when bound to a multivalent target like polysaccharide we assume it can trigger substantial receptor crosslinkage. IgG1 is less characteristic for antipolysaccharide responses and has higher affinity for FcγRs, potentially triggering cellular effector functions more efficiently. While IgE is mostly considered as high-affinity antibody, class switch to IgE antibodies independent of T-cell help has been shown in humans,^25^ suggesting its contribution to TI responses without affinity maturation and somatic hypermutation. In mice direct switching to IgE following TI stimulation was also observed, and low-affinity IgE was shown to be protective against anaphylaxis.^26^ Effector functions triggered by TI antibody responses can thus range from silent uptake to robust phagocytosis, the response being modulated by immune complex avidity and quality and extent of receptor engagement (Figure 3a).

### TD responses

T cells provide costimuli for B cells, enabling affinity maturation and class-switching. TD responses are thus characterized by non-IgM isotypes—such as IgA, IgG and IgE—and increased affinity to target (Figure 3b). Higher affinity favors the generation of higher order complexes, as the longer duration of interactions allows several different antibodies to a single target with multiple epitopes. TD responses are characteristic for protein-specific responses and protein epitopes are usually not repetitive but diverse. A polyclonal response targeting different epitopes will thus favor the generation of high avidity complexes with higher receptor-crosslinking potential. Granulocytes can arm themselves with antibodies binding to their high-affinity receptors, FcαRI,^27^ FcγRI^28, 29^ and FcεRI, and degrade target antigen following receptor crosslinkage, phagocytosis or exocytosis.

The majority of human IgE arises from class-switched B cells already undergone somatic hypermutation.^30^ The affinity of allergen-specific IgE in the nano- to picomolar *K*_D_ range has been found to correlate with cellular effects induced by antigen binding^31^ and allergy phenotype has also been related to IgE affinity.^32^ Factors promoting receptor crosslinkage, such as increased avidity due to binding to multiple epitopes and relative content of specific to total IgE also enhanced degranulation of basophil granulocytes.^33^ TD responses are more specifically targeted and highly efficient in general, as a consequence of affinity maturation and the quality of Fc receptors and effector cells the immune complexes are directed to, via isotype switching.

## Complement-mediated effector functions

**Figure 4:**
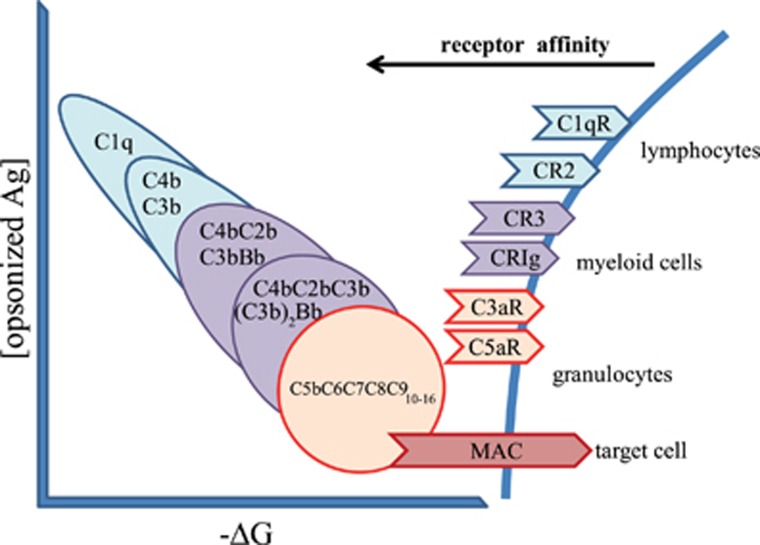
Effector functions of the complement system. Innate homeostatic regulation by the complement system employ avidity effects. Initiators of the classical and lectin pathways are oligomeric molecules with multiple binding sites. Attachment to the target by more than one arm triggers associated serine proteases and cleave complement proteins to initiate a cascade. Higher-order complexes bound to the target cell can release chemotactic substances and form a membrane attack complex (MAC) with lytic function.

The complement system is a phylogenetically ancient homeostatic system, predating the appearance of adaptive immunity.^34^ Accordingly, it can function independent of antibodies but it is also regulating and is regulated by the adaptive immune system.^35, 36^ From our point of view, an important aspect of complement-mediated immunity is that we can insert the whole system into the same framework as we did with antibodies (Figure 4). Recognition elements of the system are multivalent promiscuous binders,^37^ just as natural IgM. On the other hand, the complement system has numerous receptors and regulators, which are able to fine-tune effector functions.^38^ The classical pathway of complement can be initiated by C1q, a hexameric molecule, which binds antibodies as well as numerous self and non-self molecules.^39, 40^ The lectin pathway is triggered by mannan-binding lectin, ficolins and other multimeric molecules.^41^ Less specific recognition is coupled to avidity effects by allowing multiple binding to repetitive patterns by all these oligomeric molecules. While adaptive immunity needs time (days) to evolve and adapt to the antigen and tune the response, the complement system proceeds immediately to the next stages under appropriate conditions. Target antigen molecules bound by pattern recognition molecules can be swiftly and silently removed without further propagation of the cascade, the best example being the removal of apoptotic debris.^42^ Proteases attached to the recognition molecules can trigger proteolytic cleavage of further complement components, depending on the stability—as reflected by Δ*G*—of the complex. Covalent binding of C4b—a general feature of thioester-containing proteins^43^—further stabilizes the complex and provides ligand for other homeostatic receptors like CR3 and CR4.^44^ In the absence of inhibitory and regulatory molecules, numerous complement C3 breakdown products are generated, leading to the formation of enzymatically active C3 and C5 convertases (Figure 4). Cleavage of C3 and C5 also generates fragments with anaphylatoxic properties acting on C3aR and C5aR, recruiting and activating leukocytes. These substances can trigger the degranulation of basophil and eosinophil granulocytes, and mast cells, leading to massive systemic effects.^45^ Finally, a membrane-disrupting superstructure (membrane attack complex) may form from the assembled complement components, lysing target cells.

Thus, a sequence of events starting with avidity-mediated binding, assembly of structures with decreasing free energy and more robust cellular effects is observed, exactly as for antibodies, the difference being the lack of affinity and specificity maturation and a much faster flow of events (Figure 4).

## Interpretation of effector functions as degrees of antibody homeostasis

So far, we have outlined a scheme wherein antibodies with a range of binding affinities for antigen possess specific Fc receptors with a corresponding range of affinities. The numbers suggest that varying serum concentrations of ligands (antibodies or immune complexes) will saturate their receptors to various extents, guaranteeing a continuous homeostatic removal of antibodies for degradation. The concentration of serum IgM, and of IgA and IgG subclasses are all in the micromolar range ^46^ ensuring the binding to appropriate receptors with *K*_D_ in this range. As the apparent *K*_D_ of both immune complex formation and receptor binding decreases, the mean lifetime of immune complexes increases (Figure 2), leading to an increased chance of surface capture and internalization of bound antigens. Both of these processes are important for the development of an immune response: cell surface presentation of intact antigen is critical for B-cell development, while internalization followed by enzymatic processing and major histocompatibility complex association is required for T-cell development and activation. As the number of recruited and engaged receptors increases, the ensuing cellular responses are also tuned up. Receptor functions induced by signaling motifs in the polypeptide chains and by associated signaling molecules are effectively triggered when receptors are crosslinked by antigen-bound antibodies or multivalent antigens binding to surface-bound antibodies.^47^ This crosslinkage triggers robust cellular responses as determined by the extent of crosslinkage, identity of the cell and coupling of the receptor to the cell.^48^

FcμR are highly expressed by B cells, allowing the internalization and degradation of circulating IgM molecules^22^ as well as the presentation of bound antigen for other B cells.^1^ An IgG receptor with inhibitory signaling motif, FcγRII, is also expressed by B cells, potentially allowing the surface presentation of antigen to other B cells, as has been observed for dendritic cells.^49^ Compared to lymphocytes, myeloid cells are more potent regarding degradatory effector functions; these cells also express FcγRIII and FcγRI, along with activatory isoform of FcγRII, and can carry out a wide range of regulatory and effector functions mediated by IgG isotypes.^13^ The receptor for IgA, FcαRI has been shown to mediate inhibitory or activatory effects depending on the extent of receptor crosslinking.^50, 51^ Systemic level inflammatory reactions leading to anaphylaxis can be triggered by IgG or the anaphylatoxins C3a and C5a.^52^ The exceptionally high affinity of IgE to both its target and FcεRI can lead to highly stable antigen–antibody–receptor complexes and trigger degranulation of effector cells.^33^

**Figure 5:**
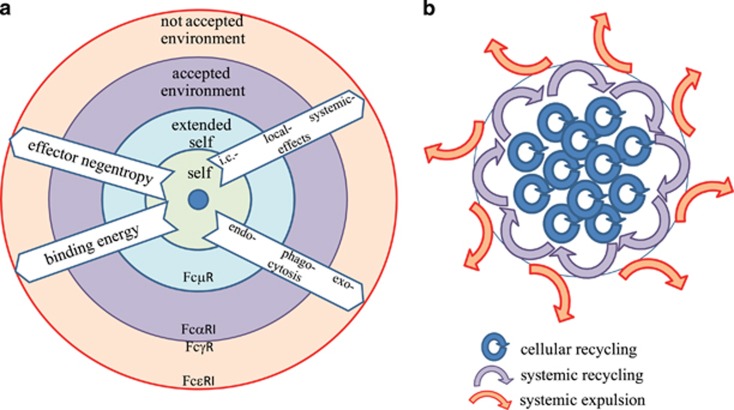
Antibody-mediated immune effector functions related to layers of immunological self. (**a**) The generation of immune complexes with greater mass correlates with a greater loss of free energy and loss of entropy. As the entropy decreases, the effector functions become more pronounced: intracellular lysosomal degradation shifts to cell activation and degranulation, with death of the effector and/or target cells. (**b**) Homeostatic function of immune effector mechanisms represented as the flow of molecular material. Effector functions mediated via cellular receptors range from local uptake and recycling of immune complex material, through systemic degradation via recruitment of cells and lysosomal degradation, to expulsion and cellular destruction via inflammatory reactions. These events are positioned to reflect immunological layers of recognition, the circle representing our immunological border. i.c., intracellular

Examination of the events following antibody binding to antigen using our proposed layers of immunological reactivity reveals that the further we leave behind immunological self the greater the energy of complex formation (Figure 5a). This is accompanied by a reduction in the number of potential microstates the involved molecules can take up, meaning the complexes becoming more and more rigid, characterized by decreased intrinsic entropy or in other words increased negentropy. Along with this increased negentropy we observe more and more pronounced molecular degradation by enzymatic processes, shifting endocytosis through phagocytosis to exocytosis. Accordingly, silent intracellular events aimed at recycling of molecular material are first replaced by enhanced local clearance activity and further on by systemic inflammatory effects induced by the release of various mediators (Figure 5b).

## Conclusions

In two previous^1, 2^ and the present papers we have outlined a general scheme for the homeostasis of antibodies. The most important message of these papers is that humoral adaptive immunity can be viewed as a physical system responsible for regulated degradation of organic material. The biophysical role of adaptive humoral immunity is to drive the system towards Global Antibody Equilibrium, a state when concentrations of free antibody, free antigen and bound antibody–antigen complexes are all equal to each other and also to the *K*_D_ of the interaction. The immunological role is to use qualitative signals of innate immunity for setting the apparent *K*_D_ for each interaction, thereby determining fate and lifetime of the target molecules. The cell biological role is to remove antibodies and bound antigen, and degrade these molecules with appropriate efficiency. The whole system maintains body integrity by setting and stabilizing molecule concentrations over several orders of magnitude (Figure 1), depending on the quality and quantity of the molecules. This range of concentrations, affinities and binding energies is placed exactly where reversible macromolecular interactions occur. Once molecules exit this scale of interactions irreversible reactions take place: molecules are degraded in enzymatic compartments of various sizes, allowing the reuse of organic material.

After all, we think that the word immunity does not fully describe the function of the adaptive immune system, as it comes from the latin 'exempt, protected'. The adaptive humoral immune system, owing to its ability of cellular evolution via genetic diversification, is an ingenious physiological molecular balance keeper of multicellular organisms, a system for the global regulation of all molecules, a continuum of recognition, complex formation and complex removal being present from 'silent' self-maintenance to 'loud' non-self expulsion. It is the qualitative signals of innate immune system, which superimposed on this physiological system render it suitable for protective purposes. Maintenance of self and expulsion of non-self are basically the very same processes, only these entities being at the ends of a wide range represent very different rates and extents of degradation. Indeed, it seems intuitive that molecules that persist for long or are continuously generated constitute an organism, while those that do not last cannot be considered a part of it. Control of the quantity and lifetime of molecules and molecular assemblies practically defines an organism—this is achieved by the humoral adaptive immune system.
